# DNA methylation of antisense noncoding RNA in the INK locus (ANRIL) is associated with coronary artery disease in a Chinese population

**DOI:** 10.1038/s41598-019-51921-3

**Published:** 2019-10-25

**Authors:** Chen-Hui Zhao, Hai-Tao Cao, Jing Zhang, Qiao-Wei Jia, Feng-Hui An, Zhao-Hong Chen, Li-Hua Li, Lian-Sheng Wang, Wen-Zhu Ma, Zhi-Jian Yang, En-Zhi Jia

**Affiliations:** 1 Department of Cardiovascular Medicine, the First Affiliated Hospital of Nanjing Medical University, Nanjing, 210029 Jiangsu Province China; 2 Department of Cardiovascular Medicine, the Friendship Hospital of Ili Kazakh Autonomous Prefecture, Yining, 835000 Xinjiang China; 3 Department of Cardiovascular Medicine, the Affiliated Changzhou No.2 People’s Hospital of Nanjing Medical University, Changzhou, 213000 Jiangsu Province China

**Keywords:** Gene regulation, Transcriptional regulatory elements

## Abstract

To explore the association between methylation of antisense non-coding RNA in the INK4 locus (ANRIL) and coronary artery disease (CAD) development. Methylation levels of ANRIL in 100 subjects with CAD and 100 controls were quantitatively analyzed using Sequenom MassARRAY. Kyoto Encyclopedia of Genes and Genomes (KEGG) pathway enrichment analysis was used to identify novel pathways. Our analyses indicated that 7 to 8 CpG sites within the 2^nd^ CpG island located upstream of ANRIL, also known as cyclin-dependent kinase inhibitor 2B – antisense 1 (CDKN2B-AS1), are hyper-methylated in CAD subjects compared to controls (*p* = 0.034). The 40^th^ CpG site within the 2^nd^ CpG island located upstream of CDKN2B-AS1 was methylated to a lesser extent in CAD subjects compared to controls (*p* = 0.045). Both Pearson and Spearman analyses indicated that methylation levels were significantly associated with total cholesterol (*r* = 0.204, *p* = 0.004), fasting high-density lipoprotein cholesterol (*r* = 0.165, *p* = 0.020), and fasting low-density lipoprotein cholesterol (*r* = 0.265, *p* = 0.000). KEGG pathway analysis revealed a significant enrichment of genes associated with the tumor necrosis factor (TNF) signaling pathway. Among them, CCAAT/enhancer binding protein (C/EBPβ) was identified as a key transcription factor that promotes expression of CDKN2B-AS1 through promotor interaction. DNA methylation of the ANRIL promoter was significantly associated with CAD development in our study. Our analyses suggest that C/EBPβ is a key transcription factor that promotes CDKN2B-AS1 expression by directly interacting with the gene promotor mediated by TNF signaling.

## Introduction

Cardiovascular disease is the leading cause of mortality worldwide^1^. Atherosclerosis is a primary risk factor for coronary artery disease (CAD), which is the most common cause of cardiovascular disease. CAD susceptibility is mediated by gene-environment interactions, as well as changes in gene expression via epigenetic regulation^2^. Therefore, future investigation into epigenetic biomarkers and diagnostic markers for CAD is necessary.

Antisense non-coding RNA in the INK4 locus (ANRIL), also known as cyclin-dependent kinase inhibitor 2B antisense 1 (CDKN2B-AS1), resides on chromosome 9p21, which has been reported to be closely associated with CAD^3^. As an important epigenetic mechanism, DNA methylation provides a molecular basis for understanding how the environment impacts the genome to modify lifelong CAD risk^4^. It has been reported that epigenetic changes in p15 (INK4b) methylation and ANRIL expression account for chromosome 9p21 mediated CAD development^5^. However, the potential role of DNA methylation of ANRIL in CAD development has not been reported. In this case-control study in China, we evaluated the relationship between DNA methylation of ANRIL and CAD.

## Subjects and Methods

### Study subjects

The study was performed using the case-control principle in accordance with the protocol approved by the Ethics Committee of the First Affiliated Hospital of Nanjing Medical University and the Friendship Hospital of Ili Kazakh Autonomous Prefecture in China. All enrolled subjects provided informed consent prior to the start of the study. This case-control study included 100 subjects with CAD and 100 controls without CAD from the Friendship Hospital of Ili Kazakh Autonomous Prefecture between 2011–2014. The CAD study population included 67 males and 33 females (mean age: 59.93 ± 6.42) and 67 male and 33 female age-matched control subjects (mean age: 58.59 ± 6.18). All subjects underwent coronary angiography to estimate the extent of CAD. All angiograms were evaluated by a cardiologist. Significant CAD was defined as at least one major epicardial vessel with >50% stenosis; control subjects presented with <50% stenosis according to the criteria defined by an ad hoc committee of the American Heart Association^6^. The severity of coronary atherosclerosis was determined using the Gensini scoring system^7^.

Subjects with spastic angina pectoris, heart failure, hepatic or renal disease, cardiomyopathy, adrenal dysfunction, and thyroid dysfunction were excluded from the study. Medical history and demographic data of the study population were collected using a questionnaire. Blood pressure was also recorded.

### Laboratory measurements

Venous blood (4 mL) was drawn after 12 hours of fasting on the second day of hospitalization for use in biochemical assays. Total cholesterol (TC, mmol/L), triglyceride (TG, mmol/L), fasting blood glucose (FBG, mmol/L), high-density lipoprotein cholesterol (HDL-C, mmol/L), low-density lipoprotein cholesterol (LDL-C, mmol/L), and creatinine (CR, μmol/L) were determined using an automated autoanalyzer (AU 2700 Olympus, 1^st^ Chemical Ltd, Japan).

### Cigarette smoking and alcohol use

A standardized questionnaire was used to assess cigarette smoking and alcohol use of the study subjects. Smoking status was classified as either “never smoking” (referred to those who never smoked) or “smoking” (including both former and current smokers). Subjects who reported consuming at least 50 g/week of alcohol were considered current drinkers. Alcohol intake status was classified as either “never drinking” or “drinking” (including both former and current drinkers)^8^.

### DNA extraction and methylation analysis

Genomic DNA was extracted from whole blood samples using the Bioteke Corporation whole blood genomic DNA purification minikit (Beijing, China, #AU18016, lot number B016007017) following the manufacturer’s instructions. Purified DNA was free of protein, nucleases, and other contaminants or inhibitors. DNA purity and concentration were estimated using the NanoDropND-2000 spectrophotometer (Thermo, Wilmington, DE, USA).

**Figure 1:**
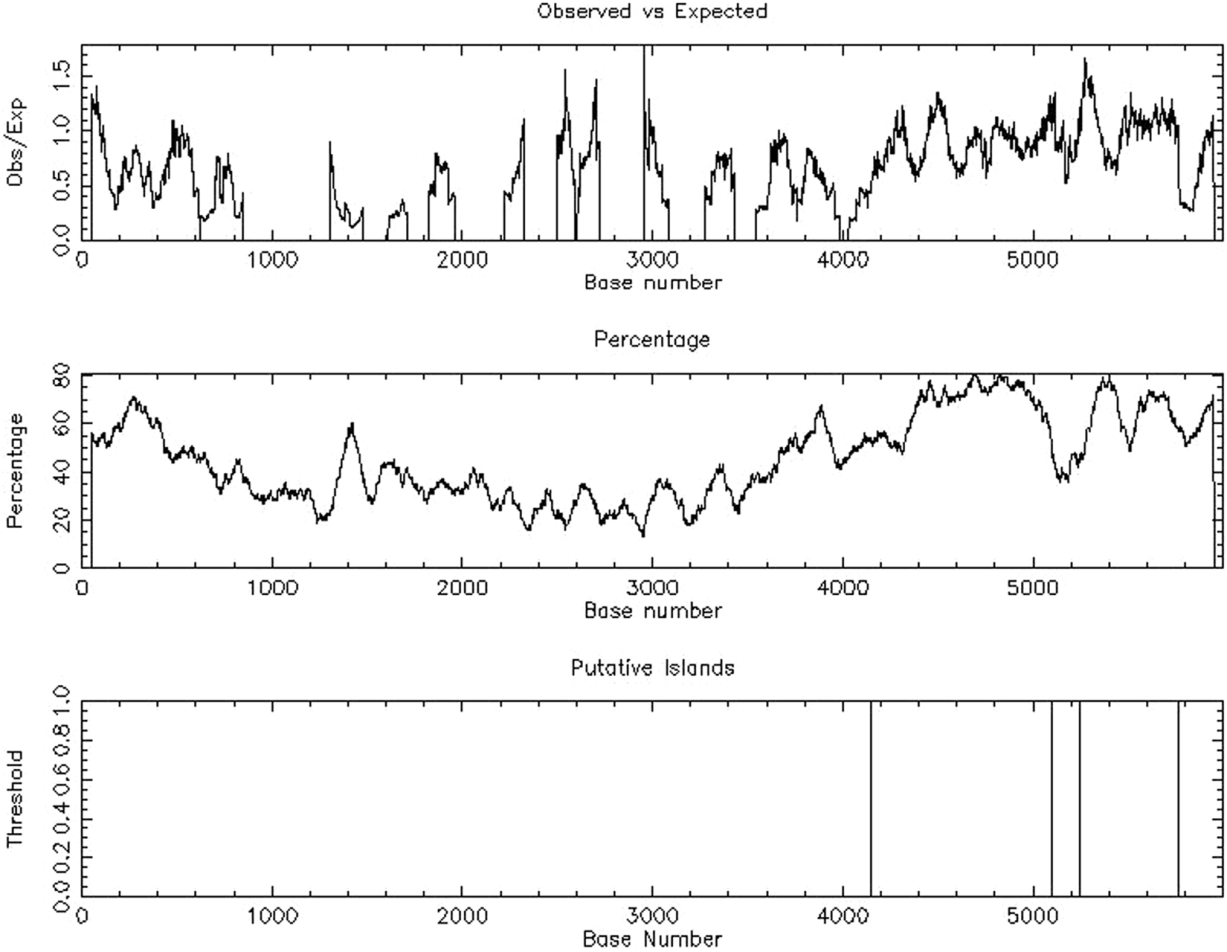
Overlapping CpG islands with the CDKN2B-AS1 promoter identified using EMBOSS software. CpG islands of unusual CG composition, EMBOSS_001 from 1 to 6,000; methylation island prediction rules: Observed/Expected ratio >0.60, Percent C + Percent G > 50.00, Length >200.

The presence of CpG islands spanning the upstream region through the downstream region (−5,000 to 1,000 bp) of the transcription start site (TSS) of the CDKN2B-AS1 gene (chr9: 21989791 ~ 21995790) was analyzed using EMBOSS (European Molecular Biology of open software http://www.ebi.ac.uk/Tools/seqstats/emboss_cpgplot/). We found two CpG islands that overlapped with the CDKN2B-AS1 promoter 6,000 bp upstream in the gDNA CDKN2B-AS1 gene sequence, a CpG island spanning from 4,149 to 5,103 bp (length 955 bp), and another CpG island spanning from 5,244 to 5,764 bp (length 521 bp) (Fig. 1). The predicted sequences of the CpG islands overlapped with the sequences of the CDKN2B-AS1 promoter.

Methylation of the TSS of CDKN2B-AS1 was quantitatively analyzed using Sequenom MassARRAY (CapitalBio), which employs matrix-assisted laser-desorption/ionization time-of-flight mass spectrometry (MALDI-TOF) and RNA base-specific cleavage. Using this system, the extracted genomic DNA was bisulfate-treated according to manufacturer’s protocol. The concentration of the bisulfate-converted DNA were determined by absorbance at 260 and 280 nm. Then, we used bisulfate-modified DNA for polymerase chain reaction (PCR) amplification. PCR primers were designed using the EpiDesigner tool (http://www.epidesigner.com). Each forward primer was tagged with a 10 mer (5′-aggaagagag-3′) to balance the PCR by adjusting for melting temperature differences, and each reverse primer had a T7-promoter tag (5′-cagtaatacgactcactatagggagaaggct-3′) for *in vitro* transcription. After PCR reaction, unincorporated deoxyribonucleotide triphosphates (dNTPs) were dephosphorylated by adding shrimp alkaline phosphatase (SAP), and SAP was then inactivated at 65 °C for 10 min^9^. The PCR reaction products were used as template in the *in vitro* transcription. After *in vitro* transcription, ribonuclease A (RNase A) was added to cleave the *in vitro* transcript. We used MALDI-TOF to analyze the products. The methylation level was expressed as the percentage of methylated cytosines over the total number of methylated and unmethylated cytosines^10^.

Two CDKN2B-AS1 primer pairs were designed using the EpiDesigner tool from Sequenom: forward: 5′-aggaagagagTTTTTGTTTTTTAGTTGGAAAGGAG-3′ and reverse: 5′-agtaatacgactcactatagggagaaggctATCCTTTATATCTAACCCATTTTTATTT-3′ (product size: 594, number of CpG’s: 43, coverage: 30); forward: 5′-aggaagagagTTTTTGTTTTTTAGTTGGAAAGGAG-3′ and reverse: 5′- cagtaatacgactcactatagggagaaggctATCCTTTATATCTAACCCATTTTTATTT-3′ (product size: 355, number of CpG’s: 28, coverage: 23). Altogether, 38 CpG sites in this region were checked. Spectra methylation ratios were generated using Epityper 1.0 (Sequenom, San Diego, CA).

### Functional annotation of CDKN2B-AS1 promoter CpG sites

**Figure 2:**
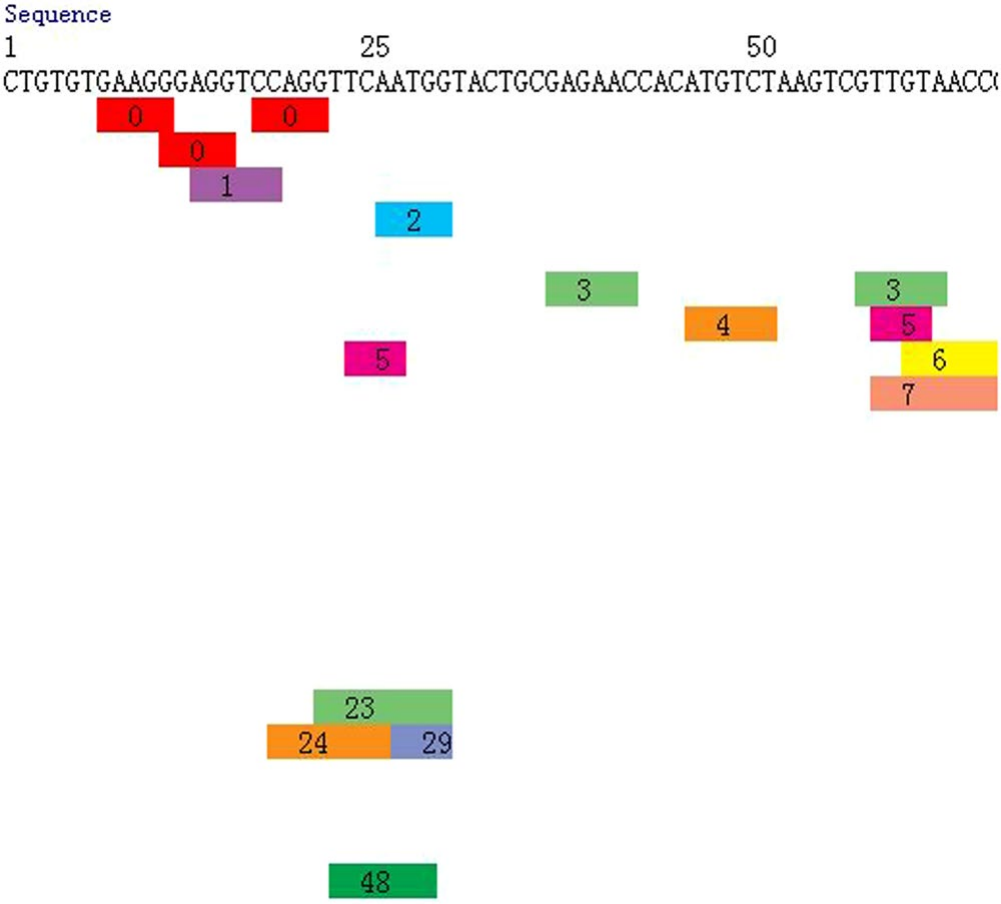
Prediction of TFBS in DNA sequences within CDKN2B-AS1 promoter CpG sites.

**Figure 3:**
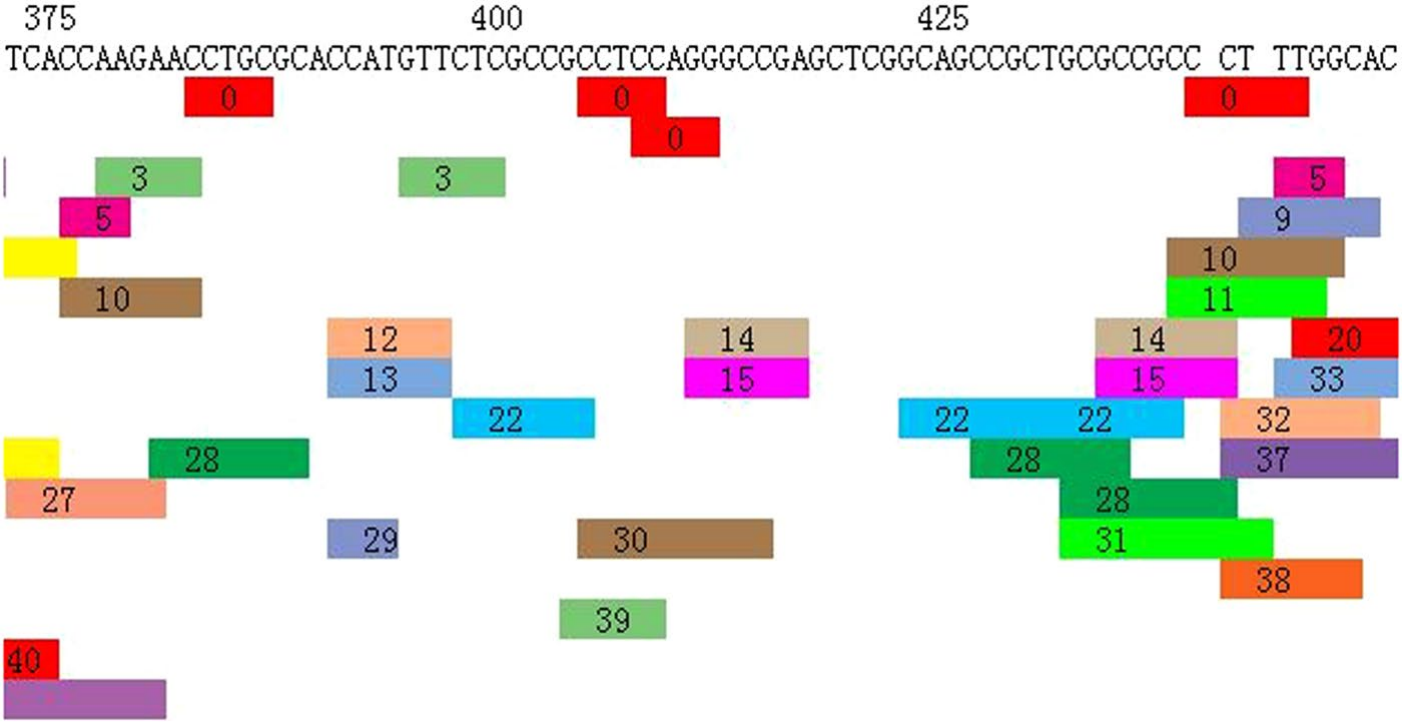
Prediction of TFBS in DNA sequences within CDKN2B-AS1 promoter CpG sites.

We used PROMO (version 8.3 of TRANSFAC) to identify putative transcription factor binding sites (TFBS) in the DNA sequences of CDKN2B-AS1’s promoter CpG sites (Figs 2 and 3). TFBS defined in the TRANSFAC database were used to construct specific binding site weight matrices for TFBS prediction^11,12^. We used Kyoto Encyclopaedia of Genes and Genomes (KEGG) enrichment analysis to identify pathways that were potentially affected by gene DNA methylation. The Database for Annotation, Visualization and Integrated Discovery (DAVID) v6.8 was used to determine ease of score differences between the CAD and control samples^13,14^.

### Statistical analysis

Data were statistically analyzed using the Statistics Package for Social Sciences (ver. 16.0; SPSS Incorporated, Chicago, IL, USA). Subjects were classified into two groups according to CAD status. Systolic blood pressure (SBP), diastolic blood pressure (DBP), TC, TG, HDL-C, LDL-C, FBG, CR, Gensini scores, and methylation levels of the CDKN2B-AS1 promoter CpG sites (except for the 7 to 8 CpG sites within the 2^nd^ CpG island and the 40^th^ CpG site within the 2^nd^ CpG island) were skewed parameters and were therefore expressed as median and quartile ranges. The Mann-Whitney U test was used to compare data parameters. Normally distributed variables, including age, methylation levels of the CDKN2B-AS1 promoter CpG sites (7 to 8 CpG sites within the 2^nd^ CpG island and the 40^th^ CpG site within the 2^nd^ CpG island) are presented as mean ± standard deviation (SD), and comparisons were analyzed using the independent-samples t test. Categorical variables, including gender, smoking status, and drinking status, were compared between groups using chi-squared analysis. The Spearman two-way test was used to assess the relationship between two quantitative variables, and we evaluated the prediction of methylation of the CDKN2B-AS1 promoter CpG sites and traditional risk factors for CAD using receiver operator curve (ROC) analysis^15^. Statistical significance was considered if the two-tailed *p* value was <0.05.

### Ethics approval and consent to participate

The study was performed in accordance with the protocol approved by the Ethics Committee of the First Affiliated Hospital of Nanjing Medical University and the Friendship Hospital of Ili Kazakh Autonomous Prefecture in China.

### Consent for publication

All authors have read and approved the manuscript.

## Results

### Characteristics of the study population

Table 1 presents the characteristics of the study population. A total of 100 subjects with CAD and 100 controls were enrolled in this study. As expected, significantly elevated SBP (*p* = 0.002), DBP (*p* = 0.002), and FBG levels (*p* = 0.001) were risk factors for CAD in this population. Additionally, Gensini scores (*p* = 0.000) were significantly different between CAD and controls.

**Table 1:** Clinical characteristics of the study population.

Characteristics	CADs (N = 100)	Controls (N = 100)	Statistical parameter	*P* value
Age (years)	59.9 ± 6.4	58.6 ± 6.2	1.232	0.226
Gender (F/M)	33/67	33/67	0.000	1.000
SBP	130 (120–140)	120 (110–130)	−3.123	0.002
DBP	80 (73–80)	80 (70–80)	−3.080	0.002
Smoking status (y/n)	47/53	39/61	1.306	0.317
Drinking Status (y/n)	88/12	84/16	0.664	0.542
TC	4.91 (4.10–5.68)	4.62 (4.05–5.39)	−1.585	0.113
TG	1.76 (1.17–2.41)	1.54 (1.07–2.16)	−1.244	0.214
HDL-C	1.35 (1.09–1.68)	1.41 (1.24–1.64)	−1.219	0.223
LDL-C	3.00 (2.40–3.62)	2.79 (2.16–3.40)	−1.731	0.083
FBG	5.34 (4.59–7.03)	4.88 (4.49–5.27)	−3.251	0.001
CR	70.00 (62.00–77.60)	67.00 (60.00–75.45)	−1.475	0.140
Gensini scores	84.50 (63.25–107.50)	0.00 (0.00–0.00)	−13.061	<0.001

Data are summarized by either mean ± standard deviation or 50^th^ (25^th^/75^th^) percentiles for continuous variables and N_1_/N_2_ for binary variables. CAD, coronary heart disease; TC, total cholesterol; HDL-C, fasting high-density lipoprotein cholesterol; LDL-C, fasting low-density lipoprotein cholesterol; TG, triglyceride; FBG, fasting blood glucose; CR, creatinine.

### Methylation of CDKN2B-AS1 in the study population

**Figure 4:**
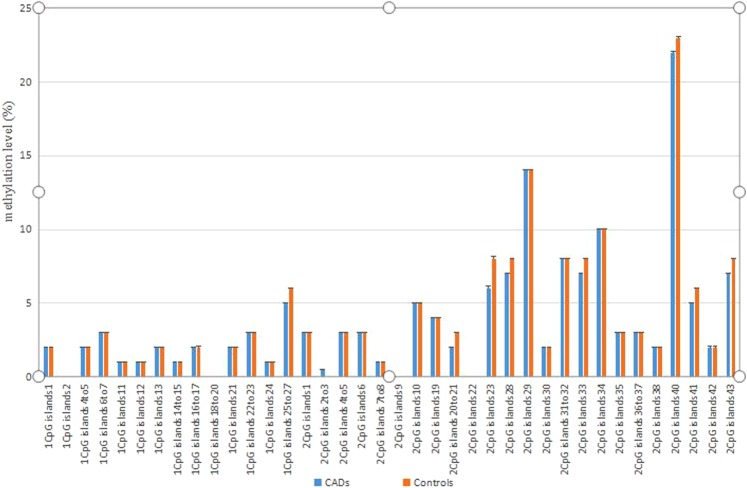
Evaluation of promoter methylation of ANRIL.

To further explore the possible molecular mechanism of CAD, we assessed DNA methylation of the CDKN2B-AS1 promoter region in CAD and control subjects. We identified 38 CpG sites upstream of the CDKN2B-AS1 gene that were methylated in the CAD and control samples using Sequenom EpiTYPER MassArra. As shown in Table 2 and Figs 4, 7 and 8 CpG sites within the 2^nd^ CpG island located upstream of CDKN2B-AS1 were hyper-methylated in CAD subjects compared to the matched controls (*p* = 0.034). The 40^th^ CpG site within the 2^nd^ CpG island located upstream of CDKN2B-AS1 was methylated to a lesser, but still significant, extent in CAD subjects compared to the matched controls (*p* = 0.045).

**Table 2:** Methylation status of ANRIL in the study population.

Characteristics	CADs (N = 100)	Controls (N = 100)	Statistical parameter	*P* value
1CpG islands1	0.02 (0.02–0.02)	0.02 (0.02–0.02)	−0.024	0.981
1CpG islands2	0.00 (0.00–0.00)	0.00 (0.00–0.00)	−0.304	0.761
1CpG islands4to5	0.02 (0.01–0.03)	0.02 (0.01–0.03)	−0.163	0.871
1CpG islands6to7	0.03 (0.02–0.03)	0.03 (0.02–0.03)	−0.934	0.350
1CpG islands11	0.01 (0.01–0.01)	0.01 (0.01–0.01)	−1.491	0.136
1CpG islands12	0.01 (0.01–0.01)	0.01 (0.003–0.01)	−1.491	0.136
1CpG islands13	0.02 (0.02–0.02)	0.02 (0.02–0.02)	−0.024	0.981
1CpG islands14to15	0.01 (0.01–0.01)	0.01 (0.01–0.01)	−1.808	0.071
1CpG islands16to17	0.02 (0.01–0.04)	0.02 (0.01–0.04)	−0.716	0.474
1CpG islands18to20	0.00 (0.00–0.01)	0.00 (0.00–0.01)	−0.195	0.846
1CpG islands21	0.02 (0.02–0.02)	0.02 (0.02–0.02)	−0.024	0.981
1CpG islands22to23	0.03 (0.02–0.03)	0.03 (0.02–0.03)	−0.930	0.350
1CpG islands24	0.01 (0.01–0.028)	0.01 (0.01–0.02)	−1.035	0.300
1CpG islands25to27	0.05 (0.05–0.06)	0.06 (0.05–0.07)	−1.904	0.057
2CpG islands1	0.03 (0.02–0.5)	0.03 (0.02–0.5)	−0.776	0.438
2CpG islands2to3	0.005 (0.00–0.01)	0.00 (0.00–0.01)	−0.500	0.617
2CpG islands4to5	0.03 (0.02–0.04)	0.03 (0.02–0.05)	−0.659	0.510
2CpG islands6	0.03 (0.02–0.05)	0.03 (0.02–0.05)	−0.776	0.438
2CpG islands7to8	0.01 (0.00–0.02)	0.01 (0.00–0.01)	−2.119	0.034
2CpG islands9	0.00 (0.00–0.01)	0.00 (0.00–0.01)	−0.496	0.620
2CpG islands10	0.05 (0.03–0.07)	0.05 (0.03–0.07)	−0.015	0.988
2CpG islands19	0.04 (0.03–0.05)	0.04 (0.03–0.05)	−0.741	0.459
2CpG islands20to21	0.02 (0.01–0.05)	0.03 (0.01–0.04)	−0.073	0.942
2CpG islands22	0.00 (0.00–0.01)	0.00 (0.00–0.01)	−0.601	0. 548
2CpG islands23	0.06 (0.00–0.13)	0.08 (0.01–0.19)	−1.794	0.073
2CpG islands28	0.07 (0.06–0.09)	0.08 (0.06–0.09)	−0.457	0.648
2CpG islands29	0.14 (0.13–0.16)	0.14 (0.13–0.17)	−0.448	0.654
2CpG islands30	0.02 (0.01–0.04)	0.02 (0.01–0.04)	−0.083	0.934
2CpG islands31to32	0.08 (0.06–0.12)	0.08 (0.06–0.11)	−0.124	0.901
2CpG islands33	0.07 (0.06–0.09)	0.08 (0.06–0.09)	−0.457	0.648
2CpG islands34	0.10 ± 0.05	0.10 ± 0.05	0.885	0.377
2CpG islands35	0.03 (0.02–0.04)	0.03 (0.02–0.04)	−0.247	0.805
2CpG islands36to37	0.03 (0.02–0.04)	0.03 (0.02–0.05)	−0.659	0.510
2CpG islands38	0.02 (0.01–0.04)	0.02 (0.01–0.04)	−0.083	0.934
2CpG islands40	0.22 ± 0.05	0.23 ± 0.05	2.017	0.045
2CpG islands41	0.05 (0.03–0.06)	0.06 (0.03–0.07)	−1.056	0.291
2CpG islands42	0.02 (0.01–0.15)	0.02 (0.01–0.15)	−0.909	0.363
2CpG islands43	0.07 (0.06–0.09)	0.08 (0.06–0.09)	−0.457	0.648

**Figure 5:**
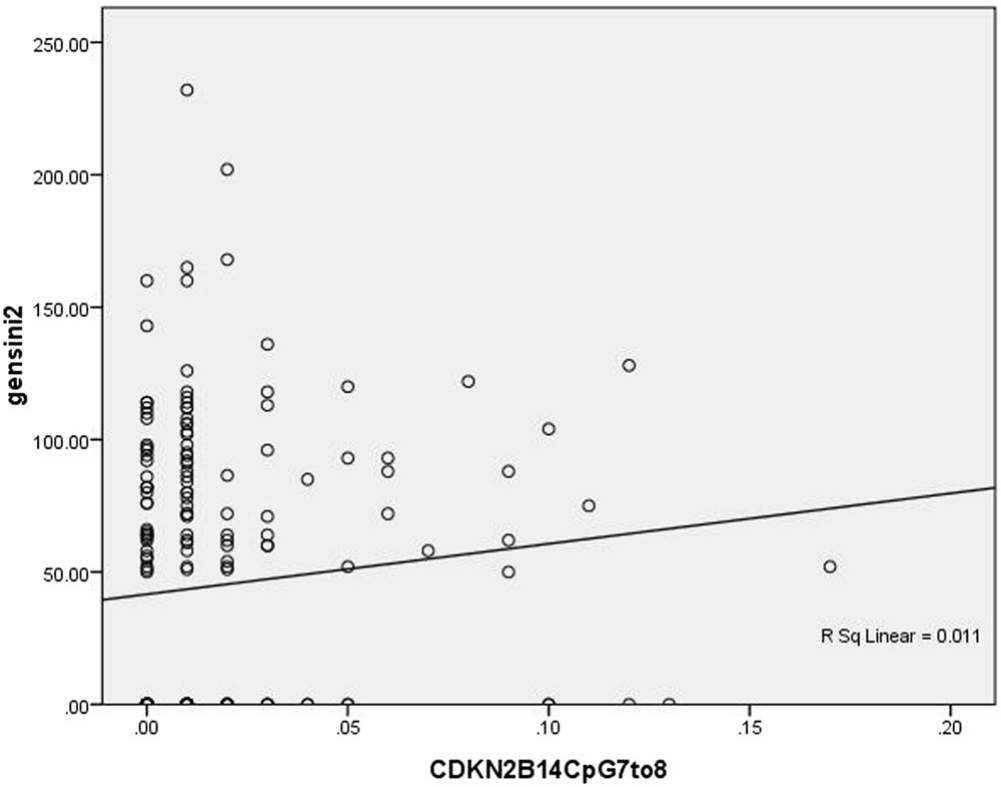
Scatter plots of Spearman correlations between Gensini Scores and ANRIL methylation of 2CpG islands 7 to 8.

**Figure 6:**
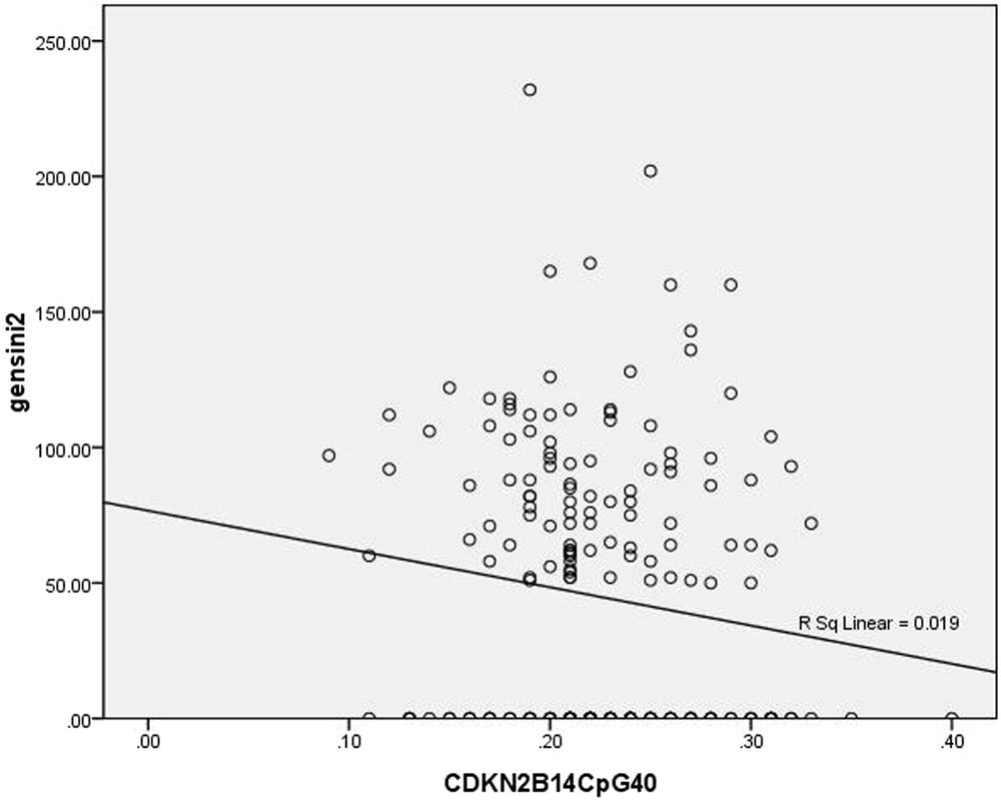
Scatter plots of Spearman correlations between Gensini Scores and ANRIL methylation of 2CpG islands 40.

**Figure 7:**
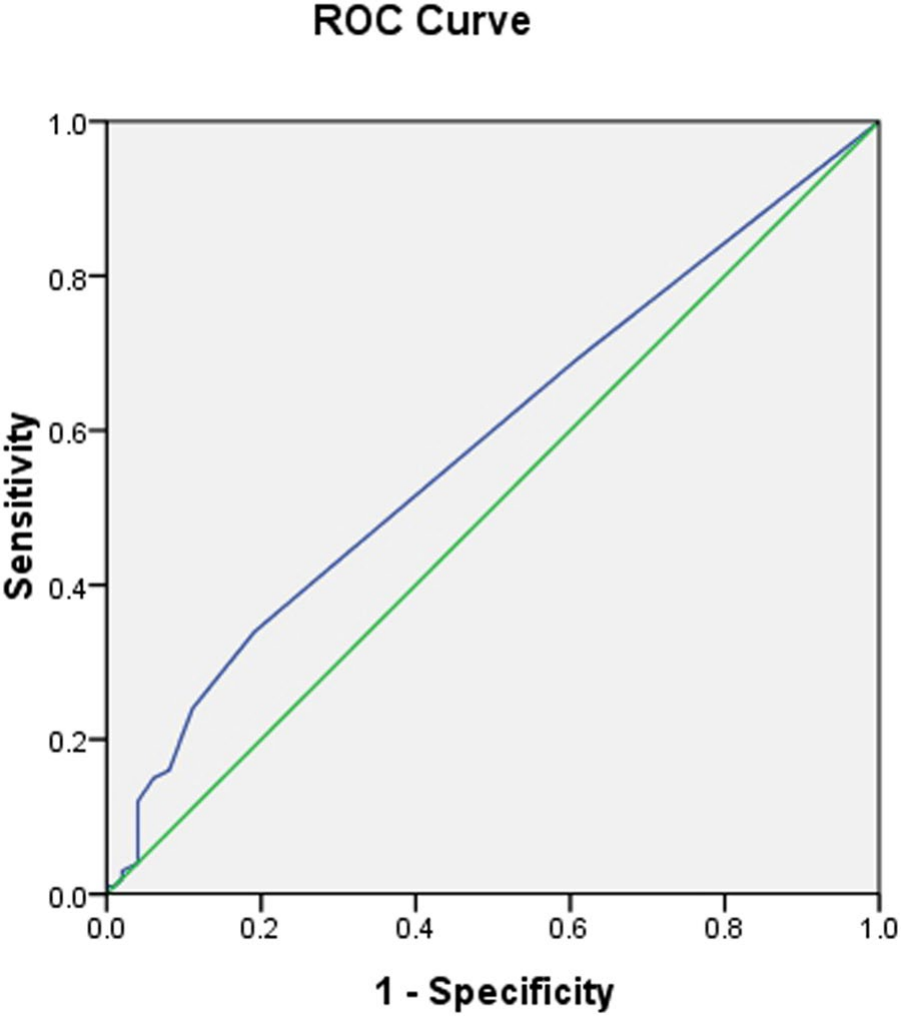
The receiver operating characteristic curve for the predictive efficacy of 2CpG islands7to8 for CAD prevalence.

**Figure 8:**
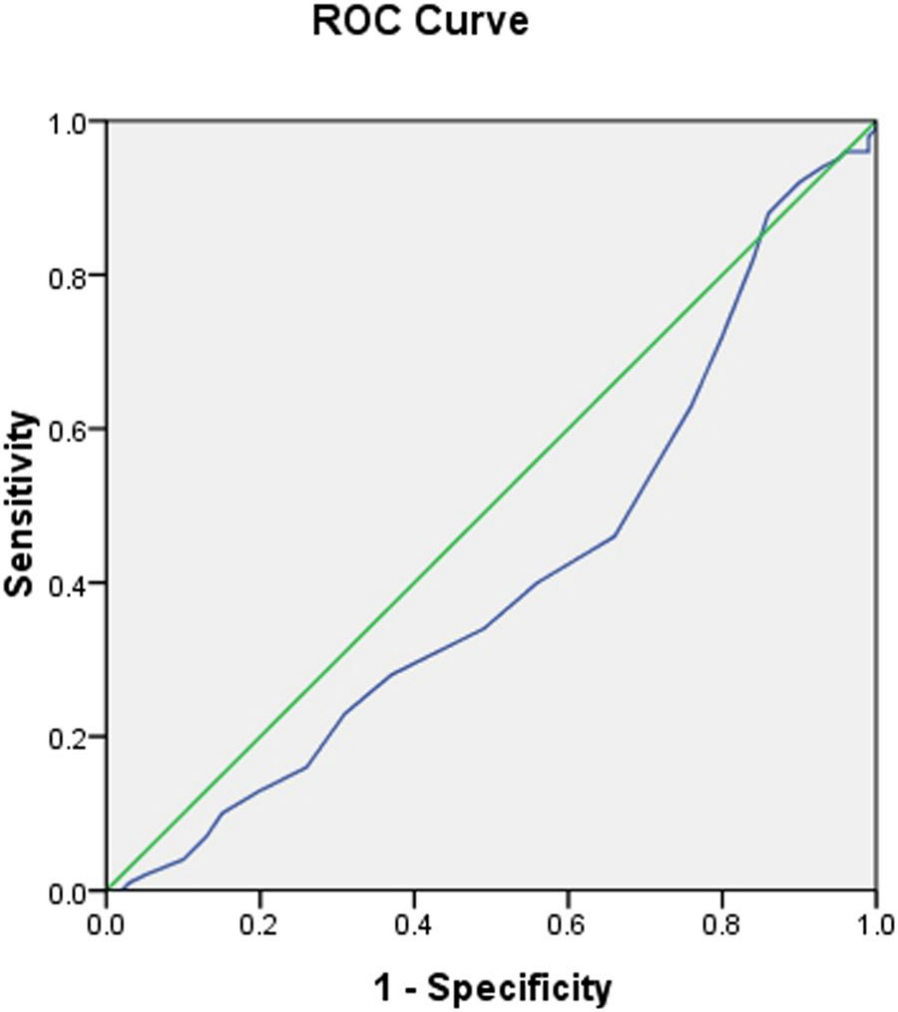
The receiver operating characteristic curve for the predictive efficacy of 2CpG islands40 for CAD prevalence.

### Correlation of Gensini scores with methylation of CDKN2B-AS1

As shown in Table 3, Spearman correlation analysis indicated that Gensini scores were positively associated with methylation levels of 7 to 8 CpG sites within the 2^nd^ CpG island located upstream of CDKN2B-AS1 (*r* = 0.137, *p* = 0.053), and that there was a significant negative association between Gensini scores and methylation levels of the 40^th^ CpG site within the 2^nd^ CpG island located upstream of CDKN2B-AS1 in the study population (*r* = −0.166, *p* = 0.019). Figures 5 and 6 show scatter plots of Spearman correlations between Gensini Scores and ANRIL methylation.

**Table 3:** Spearman correlations between Gensini Scores and ANRIL methylation.

Variables	Relationship coefficient	*P* value
1CpG islands1	0.009	0.897
1CpG islands2	−0.031	0.665
1CpG islands4to5	0.011	0.873
1CpG islands6to7	0.063	0.373
1CpG islands11	0.087	0.221
1CpG islands12	0.087	0.221
1CpG islands13	0.009	0.897
1CpG islands14to15	−0.099	0.163
1CpG islands16to17	−0.044	0.534
1CpG islands18to20	0.012	0.864
1CpG islands21	0.009	0.897
1CpG islands22to23	0.063	0.373
1CpG islands24	0.122	0.084
1CpG islands25to27	−0.128	0.070
2CpG islands1	0.031	0.667
2CpG islands2to3	0.040	0.578
2CpG islands4to5	−0.060	0.400
2CpG islands6	0.031	0.667
2CpG islands7to8	0.137	0.053
2CpG islands9	0.048	0.502
2CpG islands10	0.027	0.701
2CpG islands19	−0.020	0.782
2CpG islands20to21	0.025	0.722
2CpG islands22	−0.029	0.686
2CpG islands23	−0.142	0.045
2CpG islands28	−0.046	0.516
2CpG islands29	−0.069	0.332
2CpG islands30	−0.026	0.712
2CpG islands31to32	−0.006	0.930
2CpG islands33	−0.046	0.516
2CpG islands34	−0.020	0.780
2CpG islands35	−0.038	0.592
2CpG islands36to37	−0.060	0.400
2CpG islands38	−0.026	0.712
2CpG islands40	−0.166	0.019
2CpG islands41	−0.073	0.305
2CpG islands42	−0.080	0.261
2CpG islands43	−0.046	0.516

### Predictors of CAD prevalence

To further explore the applicability of methylation levels of CDKN2B-AS1 as a potential diagnostic biomarker of CAD, subsequent ROC analyses were performed on the above data sets (Table 4). The AUC for predicting CAD prevalence was 0.577 for methylation of 7 to 8 CpG sites within the 2^nd^ CpG island located upstream of CDKN2B-AS1 (95% confidence interval (*CI*): 0.497–0.657, *p* = 0.062)(Fig. 7), and 0.409 for methylation of the 40^th^ CpG site within the 2^nd^ CpG island located upstream of CDKN2B-AS1 (95% *CI*: 0.329–0.489, *p* = 0.028)(Fig. 8).

**Table 4:** Receiver operating characteristic curve analyses for predicting CAD prevalence.

Variables	*AUC* (95%*CI*)	*P* value
1CpG islands1	0.507 (0.426–0.588)	0.867
1CpG islands2	0.492 (0.411–0.573)	0.852
1CpG islands4to5	0.506 (0.425–0.587)	0.886
1CpG islands6to7	0.542 (0.461–0.622)	0.310
1CpG islands11	0.563 (0.483–0.643)	0.125
1CpG islands12	0.563 (0.483–0.643)	0.125
1CpG islands13	0.507 (0.426–0.588)	0.867
1CpG islands14to15	0.454 (0.373–0.534)	0.263
1CpG islands16to17	0.476 (0.395–0.557)	0.554
1CpG islands18to20	0.513 (0.433–0.594)	0.747
1CpG islands21	0.507 (0.426–0.588)	0.867
1CpG islands22to23	0.542 (0.461–0.622)	0.310
1CpG islands24	0.549 (0.468–0.629)	0.238
1CpG islands25to27	0.436 (0.355–0.517)	0.121
2CpG islands1	0.532 (0.451–0.612)	0.443
2CpG islands2to3	0.523 (0.442–0.604)	0.577
2CpG islands4to5	0.470 (0.389–0.551)	0.465
2CpG islands6	0.532 (0.451–0.612)	0.443
2CpG islands7to8	0.577 (0.497–0.657)	0.062
2CpG islands9	0.513 (0.433–0.594)	0.745
2CpG islands10	0.510 (0.430–0.591)	0.800
2CpG islands19	0.463 (0.381–0.544)	0.364
2CpG islands20to21	0.499 (0.418–0.580)	0.978
2CpG islands22	0.493 (0.412–0.574)	0.864
2CpG islands23	0.435 (0.354–0.515)	0.114
2CpG islands28	0.493 (0.412–0.574)	0.870
2CpG islands29	0.478 (0.397–0.559)	0.597
2CpG islands30	0.507 (0.426–0.588)	0.864
2CpG islands31to32	0.496 (0.415–0.577)	0.917
2CpG islands33	0.493 (0.412–0.574)	0.870
2CpG islands34	0.478 (0.397–0.559)	0.595
2CpG islands35	0.484 (0.403–0.565)	0.704
2CpG islands36to37	0.470 (0.389–0.551)	0.465
2CpG islands38	0.507 (0.426–0.588)	0.864
2CpG islands40	0.409 (0.329–0.489)	0.028
2CpG islands41	0.458 (0.377–0.539)	0.311
2CpG islands42	0.463 (0.382–0.544)	0.369
2CpG islands43	0.493 (0.412–0.574)	0.870

### Pearson and Spearman correlations between CpG island methylation and clinical characteristics of the study population

Table 5 shows the results of both the Pearson and Spearman correlation analyses between CpG island methylation and clinical characteristics of the study population. Both the Pearson and Spearman analyses indicated that methylation of the 7 to 8 CpG sites within the 2^nd^ CpG island was significantly associated with TC (*r* = 0.204, *p* = 0.004), HDL-C (*r* = 0.165, *p* = 0.020), and LDL-C (*r* = 0.265, *p* = 0.000), and methylation of the 40^th^ CpG sites within the 2^nd^ CpG island was significantly associated with age (*r = *−0.147, *p = *0.037) and FBG (*r = *−0.178, *p = *0.012).

**Table 5:** Pearson or Spearman correlation between ANRIL methylation and clinical characteristics of the study population.

Characteristics	2CpG islands7to8		2CpG islands 40	
	Relationship coefficient	*P* value	Relationship coefficient	*P* value
Age (years)	0.058	0.418	−0.147	0.037
SBP	−0.042	0.561	−0.016	0.821
DBP	−0.011	0.882	0.040	0.576
TCH	0.204	0.004	0.096	0.176
TG	−0.010	0.888	−0.012	0.870
HDL-C	0.165	0.020	0.056	0.434
LDL-C	0.265	0.000	0.093	0.191
FBG	0.053	0.453	−0.178	0.012
CR	−0.112	0.114	0.012	0.867

Data are summarized as either mean ± standard deviation or 50^th^ (25^th^/75^th^) percentiles for continuous variables and N_1_/N_2_ for binary variables. CHD, coronary heart disease; TC, total cholesterol; HDL-C, fasting high-density lipoprotein cholesterol; LDL-C, fasting low-density lipoprotein cholesterol; TG, triglyceride; FBG, fasting blood glucose; CR, creatinine.

### KEGG pathway enrichment analyses of the CDKN2B-AS1 promoter region CpG sites

**Figure 9:**
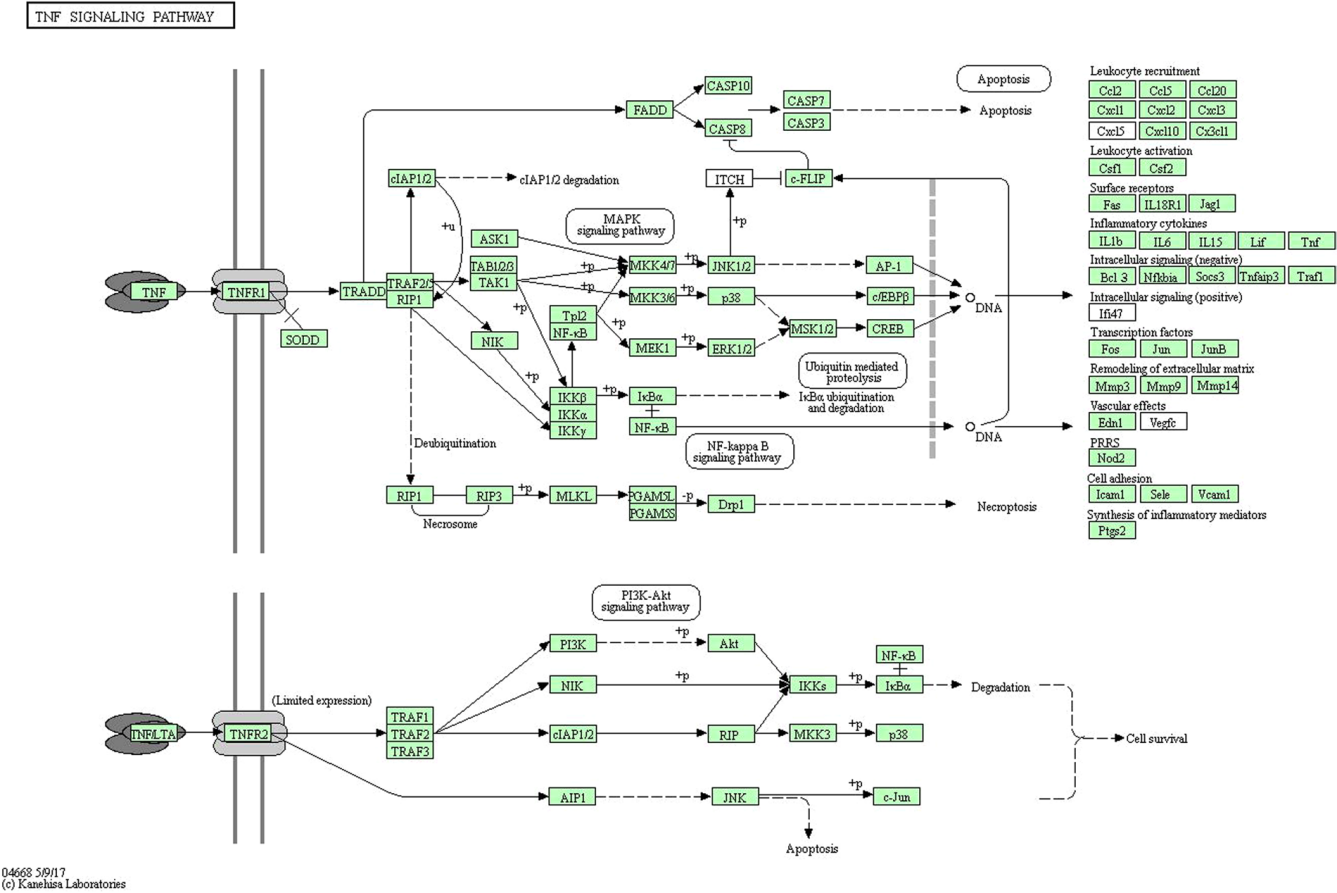
KEGG pathway enrichment analysis of methylated regions^16–18^.

We predicted 23 major putative TFBS in DNA sequences in the CDKN2B-AS1 promoter CpG sites (within the upstream region to the downstream region spanning from −30 to 30 bp of the 7 to 8 CpG sites within the 2^nd^ CpG island and the 40^th^ CpG site within the 2^nd^ CpG island). We then used KEGG pathway enrichment analyses to identify pathways possibly influenced by the genes nearest to the CpG sites in subjects with CAD compared to controls. KEGG pathway analysis revealed a significant enrichment of genes associated with the TNF signaling pathway (Fig. 9)^16–18^. Among them, CCAAT/enhancer binding protein β (C/EBPβ) was identified as a key transcription factor that could increase expression of CDKN2B-AS1 through interaction with its promoters.

## Discussion

In this case-control study of a Chinese population, we found novel associations between CAD development and methylation levels of the CDKN2B-AS1 (ANRIL) promoter region CpG sites, which were significantly correlated with blood glucose and cholesterol levels, respectively. KEGG pathway enrichment analyses suggested that the TNF signaling pathway is involved in C/EBPβ-mediated increases in CDKN2B-AS1 expression. To the best of our knowledge, this is the first study focused on the relationship between DNA methylation of ANRIL and CAD.

Genome-wide association studies (GWAS) have found that multiple single nucleotide polymorphisms (SNPs) on chromosome 9p21 are highly associated with CAD^19–26^. However, the mechanism underlying this association remains elusive. One study suggested that epigenetic changes in p15 (INK4b) methylation and ANRIL expression are associated with chromosome 9p21 related risk of CAD development^5^. Recently, a few studies have explored the association between ANRIL promoter methylation and CAD risk factors. These studies demonstrated that epigenetic regulation of ANRIL promoter methylation is associated with childhood bone development^27^, adiposity^28^, and increased arterial pulse wave velocity (PWV, a measure of arterial stiffness)^29^. Therefore, it is necessary to explore the relationship between ANRIL promoter DNA methylation and CAD development.

In the present study, we found novel associations between CAD and methylation levels of the CDKN2B-AS1 promoter region CpG sites. Furthermore, we identified significant correlations between blood glucose and cholesterol levels with ANRIL promoter methylation. These findings may partially underlie the risk associated with chromosome 9p21 and CAD. However, the exact mechanism underlying ANRIL promoter DNA methylation and CAD remains unknown. Our KEGG pathway analysis demonstrated a significant enrichment of genes associated with the TNF signaling pathway, and C/EBPβ was identified as a key transcription factor that interacts with the ANRIL promotor. A previous study used loss-of-function and chromatin immunoprecipitation approaches to show that TNF-α induced ANRIL expression^30^. C/EBPβ is a transcription factor that belongs to a class of the basic-leucine zipper proteins that has phylogenetic, structural, and functional features. A related study confirmed that C/EBPβ regulates pro-inflammatory responses^31^. Therefore, C/EBPβ may be a potential target for treating CAD. It is important to note, however, that the results from our KEGG pathway analysis should be further confirmed in functional and mechanistic studies.

Our study has limitations that should be considered when interpreting the results. First, our study was a case-control, cross-sectional design with a small sample size. Moreover, our study only provides evidence for the potential association between ANRIL promoter DNA methylation and CAD; whether changes in DNA methylation are causative of CAD pathogenesis requires further evaluation. Finally, the mechanism underlying ANRIL promoter DNA methylation and CAD was predicted by KEGG pathway analysis rather than by experimental confirmation. Therefore, additional case studies with larger sample sizes are needed to confirm our results.

## Conclusion

In summary, DNA methylation of the ANRIL promoter was significantly associated with CAD development in this Chinese study population. KEGG pathway enrichment analyses indicated C/EBPβ as a key transcription factor that promotes CDKN2B-AS1 expression mediated by TNF signaling.

## Data Availability

All data and materials have been made available.
